# Identification of an immune-related gene prognostic index for predicting survival and immunotherapy efficacy in papillary renal cell carcinoma

**DOI:** 10.3389/fgene.2022.970900

**Published:** 2022-08-29

**Authors:** Dongshan Chen, Chen Zhang, Yuanwei Zang, Wei Wang, Jiandong Zhang

**Affiliations:** ^1^ Department of Urology, Beijing Chaoyang Hospital Affiliated Capital Medical University, Beijing, China; ^2^ School of Life Science and Engineering, Handan University, Handan, China; ^3^ Department of Urology, Qilu Hospital of Shandong University, Jinan, China; ^4^ Department of Urology, Shanxi Bethune Hospital, Shanxi Academy of Medical Sciences, Taiyuan, China; ^5^ Department of Urology, Tongji Hospital, Tongji Medical College, Wuhan, China

**Keywords:** immune cell infiltration, immune-related genes, papillary renal cell carcinoma, prognostic index, immunotherapy

## Abstract

Despite considerable progress has been made in the understanding of the genetics and molecular biology of renal cell carcinoma (RCC), therapeutic options of patients with papillary renal cell carcinoma (PRCC) are limited. Immunotherapy based on immune checkpoint inhibitors (ICIs) has become a hot point in researching new drug for tumor and been tested in a number of human clinical trials. In this study, an immune-related gene prognostic index (IRGPI) was developed and provided a comprehensive and systematic analysis of distinct phenotypic and molecular portraits in the recognition, surveillance, and prognosis of PRCC. The reliability of the IRGPI was evaluated using independent datasets from GEO database and the expression levels of the genes in the IRGPI detected by real-time PCR. Collectively, the currently established IRGPI could be used as a potential biomarker to evaluate the response and efficacy of immunotherapy in PRCC.

## Introduction

Renal cell carcinoma is the third most common urological cancer, accounting for 3% of all cancers in women and 5% in men with an incidence of around 400,000 new cases worldwide (Siegel et al., 2019; Deleuze et al., 2020). Papillary renal cell carcinoma (PRCC) usually occurs in sporadic forms and is the second most common subtype of renal carcinoma, behind clear cell RCC (ccRCC) (Akhtar et al., 2019). PRCC is typically divided histologically into 2 types, type 1 (characterized by a basophilic cytoplasm and considered as a low-grade tumor) and type 2 (characterized by a bulky eosinophilic cytoplasm and pseudostratified tumor cell nuclei and considered as a high-grade tumor), which is commonly associated with higher frequency of necrosis and worse outcome (Delahunt et al., 2001; Pignot et al., 2007; Deng et al., 2019). In point of view of clinical manifestations, the patients with PRCC are characterized by hematuria, flank pain and a palpable abdominal mass, which generally appear in the late stage. Accordingly, most of the patients are diagnosed at locally advanced stages and metastatic disease (Courthod et al., 2015). Unfortunately, nearly 40 percent postoperative patients would have local recurrence and blood vessel metastasis (Courthod et al., 2015). Thus, it is absolutely critical for us to explore and develop reliable biomarkers for diagnosis and prognosis of PRCC, which may serve as a potential therapeutic target for the clinical management.

Despite some progress, therapy targeting of VEGF and mTOR signaling has an presents few clinic effect in patients with PRCC, while the efficacy of VEGFR-targeted therapies and mTOR inhibitors has been demonstrated in clinical trials in patients with ccRCC (Tannir et al., 2016). Recently, there is an increased interest in considering “targeted immunotherapy” as a perspective, effective therapeutics of solid tumors. Immunotherapy based on immune checkpoint inhibitors (ICIs) has become a hot point in researching new drug for tumor and been tested in a number of human clinical trias. At present, programmed death‐1 (PD‐1), programmed death ligand 1 (PD‐L1) and cytotoxic T lymphocyte associated protein 4 (CTLA4), which mediate critical pathway responsible of immune-tolerance against tumor cells, are the major therapeutic target of ICIs therapy (Gunturi and McDermott, 2014; Del Paggio, 2018). A single arm, non-randomized, Phase I trial confirmed the efficacy of ipilimumab, a fully human monoclonal antibody anti-CTLA4, in the improvement of the objective response rate by inducing the immunologic rejection (Yang et al., 2007). And in another investigation, PD-L1 positivity in non-clear cell RCC, especially papillary RCC cells showed a negative prognostic role, being significantly correlated with advanced grade and with shorter overall survival (Choueiri et al., 2014).

It can be seen that prognostic biomarkers based on immunology may help the clinical risk stratification and prognosis decision of patients with PRCC. In this study, we sought to identify a novel immune signature which could be used to determine the characteristic and prognosis, and could be applied as therapeutic targets for gene therapy of PRCC. We utilized the data from The Cancer Genome Atlas (TCGA) and identified an immune-related gene prognostic index (IRGPI) by weighted gene co-expression network analysis (WGCNA) with immune-related hub genes related to prognosis. We then evaluated the clinical value of the immune signature and analyzed the correlation between the signature and immune infiltration in PRCC.

## Materials and methods

### Data source and preprocessing

The entire RNA-sequencing profiling data, gene mutation information and clinical information of 321 patients with PRCC were collected from the TCGA data portal (https://tcga-data.nci.nih.gov/tcga/). The Ensembl IDs of genes was converted into a matrix of gene symbols by the Ensembl database (http://asia.ensembl.org/index.html).

Moreover, we also downloaded the raw expression data and clinical information of 34 patients with PRCC from Gene Expression Omnibus (GEO) database (http://www.ncbi.nlm.nih.gov/geo) as the external validation dataset (GSE2748). The lists of immune-related genes were downloaded from the ImmPort (https://www.immport.org/shared/home) and InnateDB (https://www.innatedb.com/) databases.

### Identification of differentially expressed immune-related genes (ir-DEGs)

We identified differentially expressed genes between normal and PRCC samples by screening criteria of *p* value >0.05 and log(fold-change) >1. Next, we took the intersection of immune-related genes and differentially expressed genes elected genes for constructing the immune‐related risk signature. The package “limma” in the statistical software R was used for this difference analysis (Xu and Wunsch, 2010).

### Gene set enrichment analysis

Gene set enrichment analysis (GSEA) was performed for analyzing the gene ontology (Subramanian et al., 2005). All the gene ontology gene sets involved in our study were obtained from Molecular Signatures Database (MSigDB) (http://software.broadinstitute.org/gsea/downloads.jsp). Kyoto Encyclopedia of Genes and Genomes (KEGG) pathway analysis was used to excavate remarkable pathways associated with differentially expressed immune-related genes. GO and KEGG were performed by clusterProfiler package of R.

Based on the 581 differentially expressed immune-related genes, we subsequently performed weighted gene co-expression network analysis (WGCNA) to identify gene modules with similar expression patterns and analyze the relationship between gene expression and clinical phenotypes (Langfelder and Horvath, 2008). Then 7 modules were identified by the pruning dimension tree as the merging threshold function at 0.25. Gene significance (GS) indicates the intensity of linear correlation between the expression of different gene modules and clinical characteristics.

### Construction and validation of the immune‐related risk signature

Univariate Cox regression analysis of these immune‐related genes was conducted to identify the immune‐related genes associated with overall survival, which were selected for follow-up study (Supplementary Table S1). The 50 selected immune‐related genes were fitted into multivariate Cox regression analysis to construct an immune-related gene prognostic signature. The formula for the prognostic score model was as followed:

Risk score = β_ir-gene-1_ × Expression _ir-gene-1_ + β_ir-gene-2_ × Expression _ir-gene-2_ + ... + β_ir-gene-n_ × Expression _ir-gene-n_.

Based on the formula above, the IRGPI risk scores of each sample were calculated and divided into high- and low-risk groups with the median risk score as the cutoff. Subsequently, Kaplan Meier analysis was used in survival analysis and Log rank method in comparison to assess the differences in overall survival (OS) between the high-risk and low-risk groups with “survival” package in R. However, due to our limited level, we have not yet retrieved a data set containing PRCC prognostic information with a sufficiently large sample size. In order to verify the prognostic value of this signature, we tested the diagnostic performance of the prognostic signature on distinguishing class 1 (corresponded to 3 histological subtypes: Type 1, low-grade Type 2 and mixed Type 1/low-grade Type 2 tumors) and class 2 (corresponded to high-grade Type 2 tumors) based on the validation cohort (GSE2748) from GEO database.

### The gene set enrichment analysis enrichment analysis, gene mutation analysis and immune infiltration analysis

Gene set enrichment analysis (GSEA) was performed to determine whether the signaling pathways showed statistically significant and concordant differences between high-risk and low-risk groups. We further performed gene mutation analysis to analyze the quantity and quality of gene mutations between two IRGPI subgroups by using the Maftools package of R. In the immune infiltration analysis, CIBERSORT (https://cibersort.stanford.edu/) was used to estimate the relative proportion of 22 types of immune cells. Then, we compared the relative proportions of 22 types of immune cells and clinicopathological features between high and low score group.

### Receiver operating characteristic curve analysis

The area under the receiver operating characteristic (ROC) curve (AUC value) obtained from ROC curve analysis was utilized to compute the sensitivity and specificity and to evaluate diagnostic efficacies of the prognostic signature in predicting patient prognosis using the R package “survival ROC”. Moreover, we performed survival analyses in two urothelial cancer cohorts treated with anti-PD-L1 and performed time-dependent ROC curve analyses to compare the prognostic value among IRGPI, tumor immune dysfunction and exclusion (TIDE), and 18-gene T cell inflamed signature (TIS) with the timeROC package of R (Ayers et al., 2017; Chen et al., 2021).

### Cell culture, RNA extraction and quantitative reverse transcription PCR (qRT-PCR)

The human proximal tubular epithelial cell line (HK-2) and human PRCC cell line ACHN were contained in our laboratory in RPMI-1640 medium (HyClone, Logan, UT, United States) containing 10% fetal bovine serum (Gibco/BRL, Grand Island, NY), 100 U/ml penicillin sodium and 100mg/ml streptomycin sulphate, 37°C, 5% CO2 incubator in the closed-culture. All cell lines were obtained from American Type Culture Collection (ATCC). Total RNA was extracted from cells using TRIzol reagent (Invitrogen, Frederick, MD, United States). RNA was converted to cDNA using standard techniques. Real-time RT-PCR was carried out using multiple kits (SYBR Premix Ex Taq, Takara Bio, DRR041A) according to the manufacturer’s instructions on CFX96 (Bio-Rad Laboratories, Hercules, United States). All primer sequences were listed in Supplementary Table S2. Statistical analysis was performed using GraphPad Prism software.

### Statistical analysis

The boxplots were conducted using the R package called “ggplot2”. Kaplan-Meier curve was used to evaluate the OS between low-risk group and high-risk group. We performed Wilcoxon tests to assess the Differences between variables. Kaplan-Meier curve was used to evaluate the OS between low-risk group and high-risk group. Varieties of risk gene expression in PCR were determined using Student’s t test. All statistical analysis in this study was applied by Perl (version 5.30.1.1, http://www.perl.org), R (version 4.0.3, https://www.r-project.org) and GraphPad Prism (version 8, https://www.graphpad.com/). *p* < 0.05 was considered significantly statistical difference.

## Result

### Identification of differentially expressed genes

**FIGURE 1:**
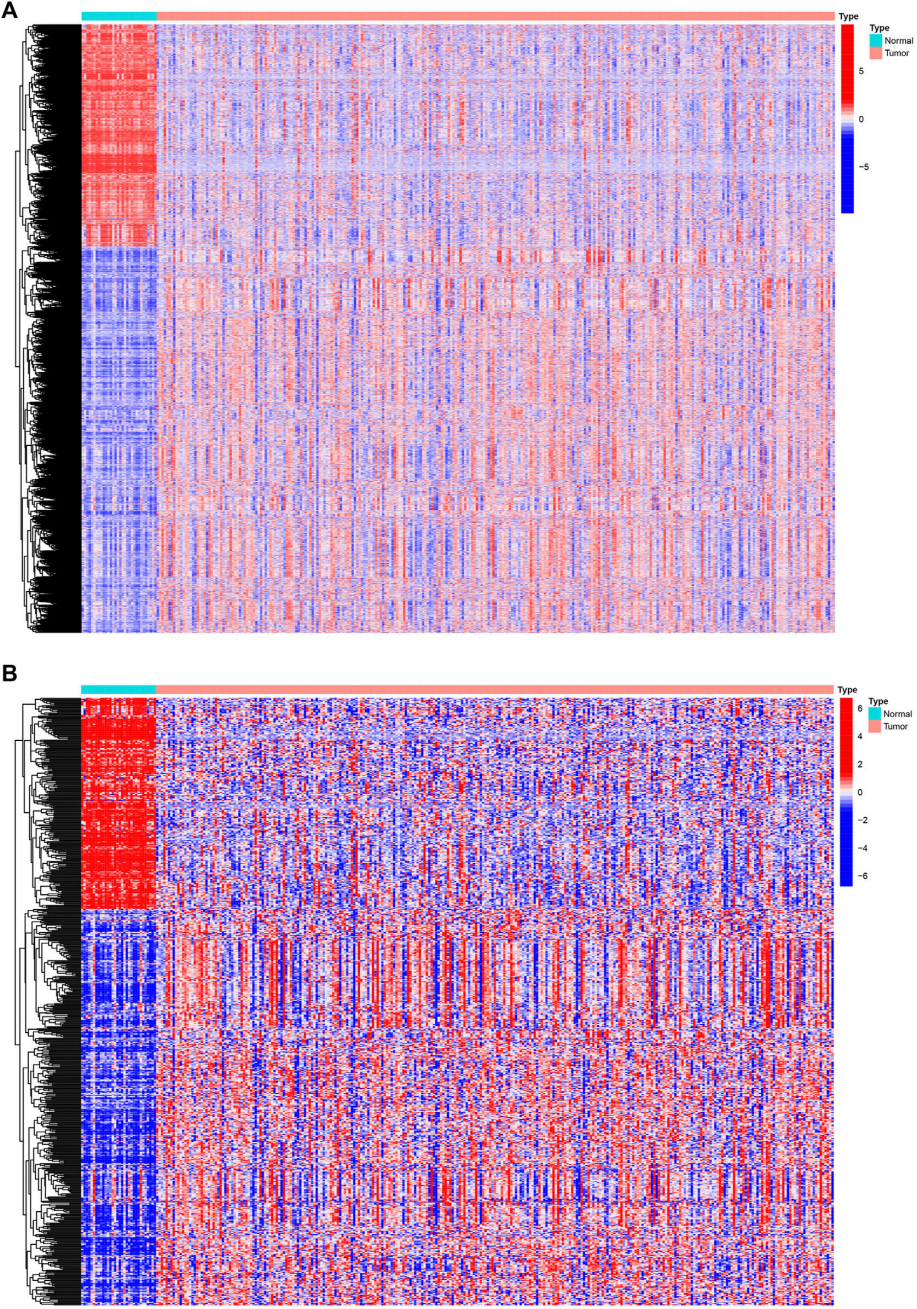
Differentially expressed IRGs. The heatmap **(A)** of differentially expressed genes in PRCC samples, compared to adjacent normal samples. The heatmap **(B)** and of differentially expressed IRGs. Red represents higher expression genes, blue represents lower expression genes, black represents same expression genes (fold change > 1.5 and *p* < 0.05). IRGs, immune-related genes; PPRC, papillary renal cell carcinoma.

We analyzed Affymetrix microarray data of all 321 PRCC cases downloaded from TCGA database and obtained the differentially expressed genes between normal and tumor samples (Figure 1A). By taking the intersection of DEGs and immune-related genes, differentially expressed immune-related genes was obtained for constructing the IRGPI (Figure 1B). Red represents higher expression genes, green represents lower expression genes.

**FIGURE 2:**
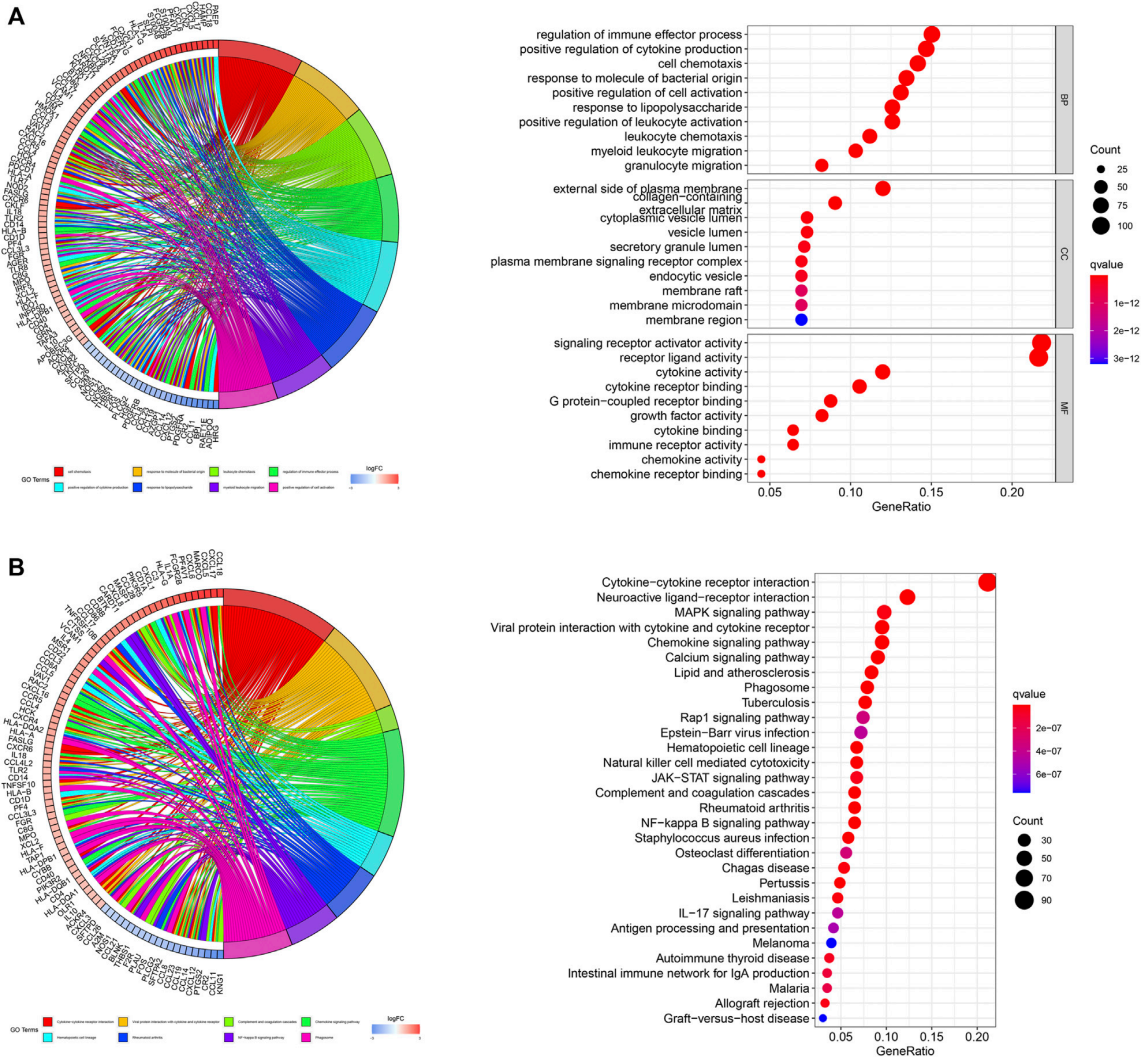
Functional enrichment analysis of differentially expressed IRGs. **(A)** Chord plot and bar graph of GO enrichment analysis. **(B)** Chord plot and bar graph of the top 30 KEGG signaling pathways. IRGs, immune-related genes; BP, biological process; CC, cellular component; MF, molecular function; KEGG, Kyoto encyclopedia of genes and genomes.

We subsequently performed functional enrichment analysis for functional annotation of the ir-DEGs. GO enrichment analysis found that these ir-DEGs were more enriched in regulation of immune effector process, positive regulation of cytokine production, cell chemotaxis, external side of plasma membrane, signaling receptor activator activity, cytokine activity and cytokine receptor binding, indicating that these ir-DEGs were closely related to intensive immune phenotype (Figure 2A). We further demonstrated the KEGG pathway enrichment analysis of the top 30 immune‐related gene ontology terms in Figure 2B, and assessed the potential function of the by ir-DEGs.

### Weighted correlation network analysis and co-expression network analysis

**FIGURE 3:**
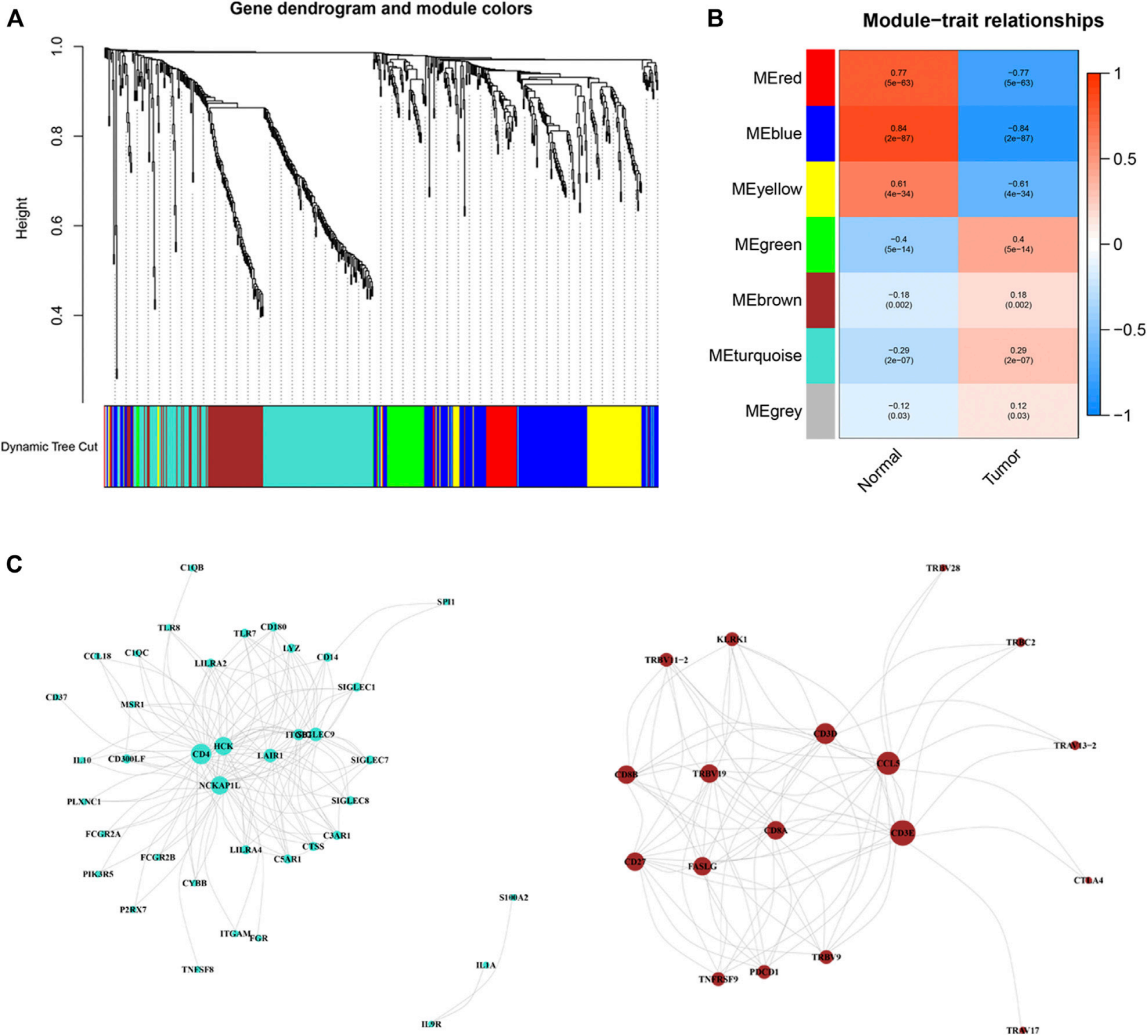
Weighted correlation network analysis and co-expression network analysis. **(A)** Dendrogram showing all differentially expressed genes clustered based on different metrics. **(B)** Heat‐map of the correlation between gene modules and the clinical phenotypes of PRCC. **(C)** Visualization of turquoise and brown modules gene networks. PPRC, papillary renal cell carcinoma.

Based on these ir-DEGs from TCGA PRAD expression profiles, WGCNA was performed to identify seven modules by the soft-thresholding power of 3 (Figure 3A). The clinical relevance of those seven modules were illustrated in Figure 3B. Afterwards, a co-expression network of these genes was constructed based on turquoise and brown modules, which were selected for further analysis (Figure 3C). The size of the nodes dictated the degree of tightness with other ir-DEGs.

### Construction and validation of the immune-related gene prognostic index

**FIGURE 4:**
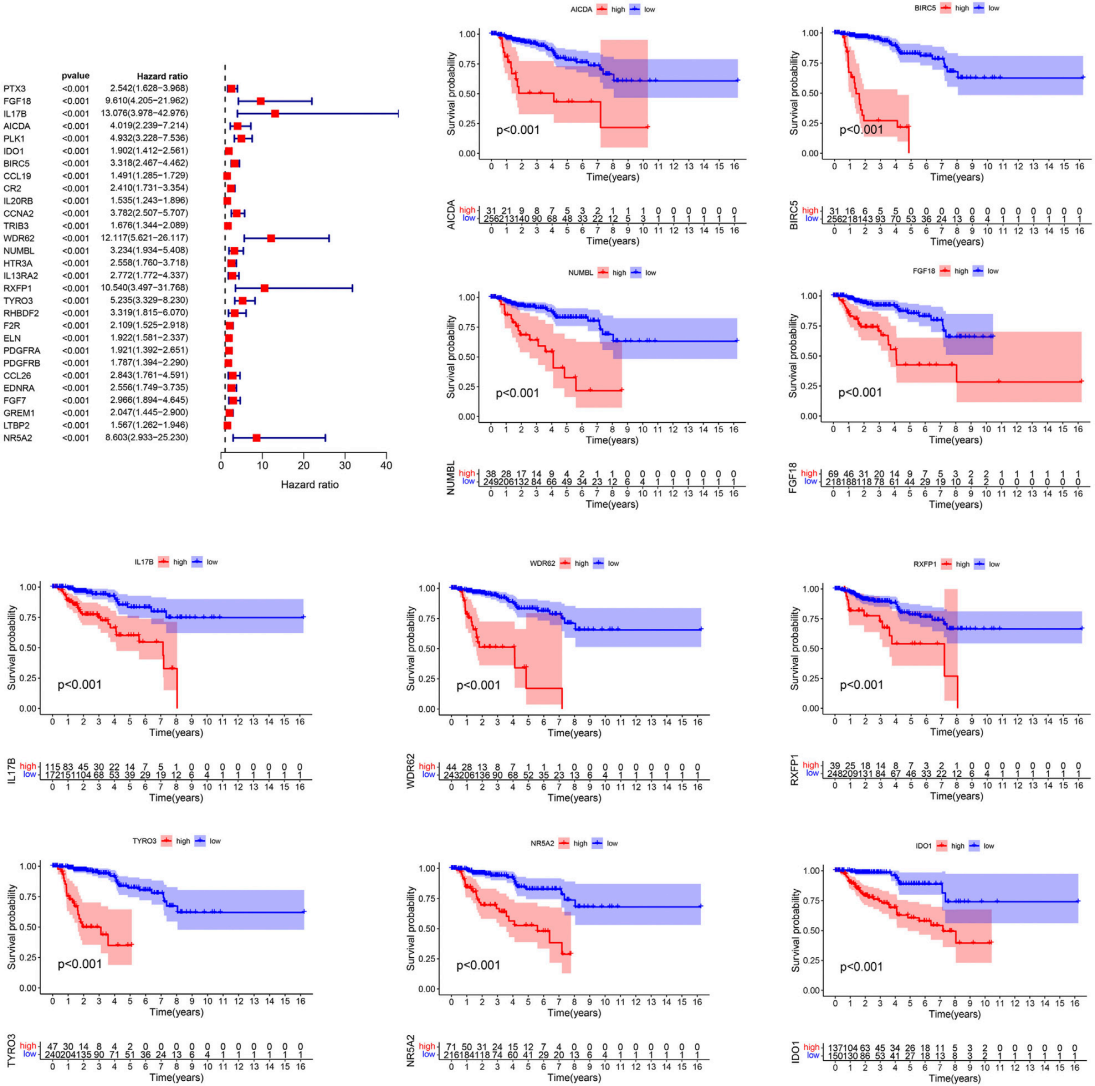
Prognostic values of survival-related IRGs. *p* values < 0.05 were considered to be statistically significant. IRGs, immune-related genes.

**FIGURE 5:**
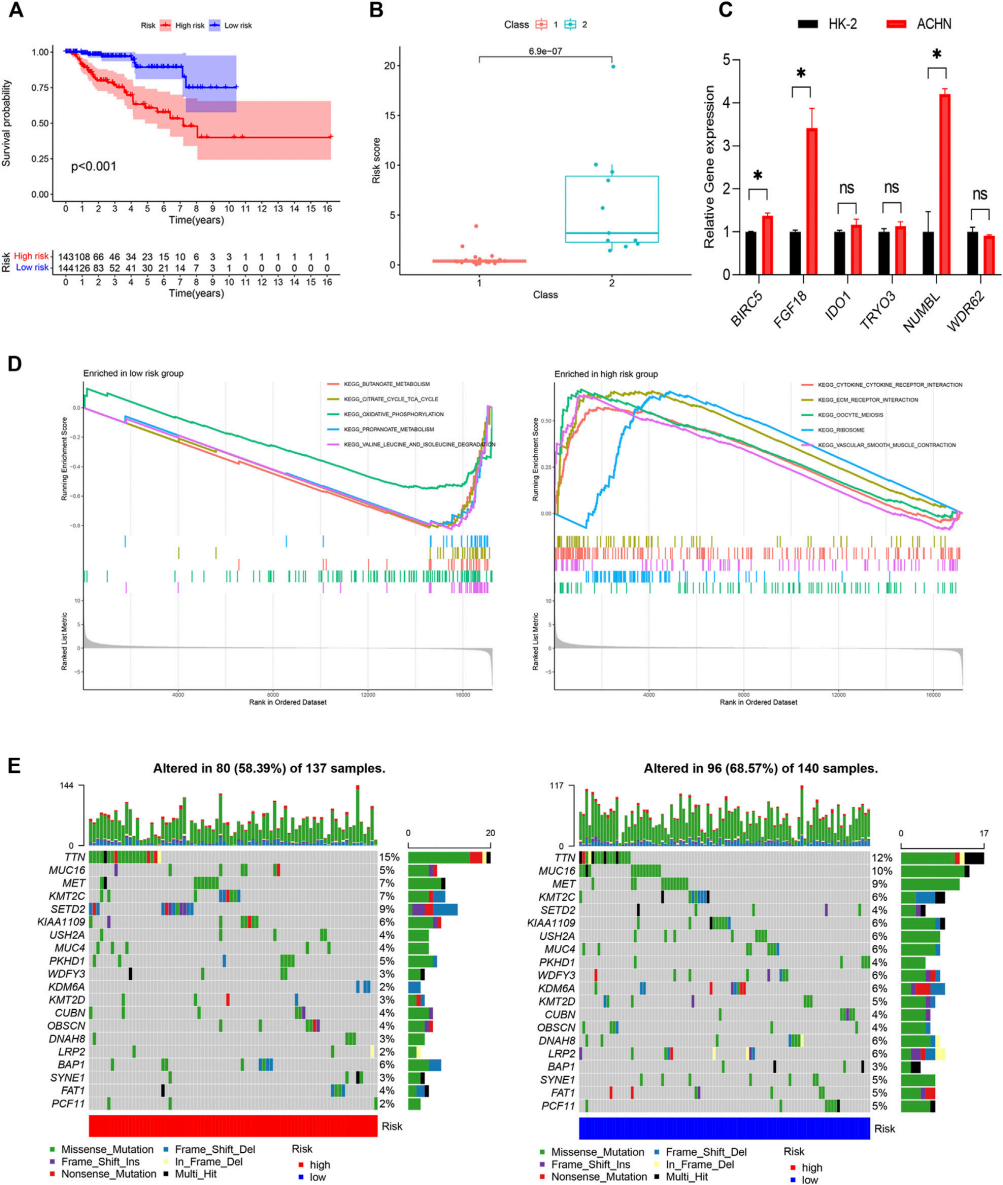
Construction of the IRGPI. **(A)** The K-M survival analysis of the IRGPI. **(B)** The relationships between IRGPI and pathological classification in PRCC cohort from GEO database. **(C)** The gene expression levels in the IRGPI were evaluated by real-time qPCR. *p < 0.05; ns, non significance. **(D)** Different gene sets enriched in the IRGPI subgroups. **(E)** Differences in the mutational status within the IRGPI subgroups. IRGPI, immune-related gene prognostic index.

The genes of the turquoise and brown modules were analyzed by univariate Cox regression, and a total of 29 genes had proven to be enormously valuable for predicting prognosis of PRCC. The resulting forest plot and survival analysis of partial genes was shown in Figure 4. The 29 survival-related genes were then subjected to multivariate Cox regression analysis to construct an IRGPI (Table 1). According to this model, all samples were divided into a high- and low-risk group with the median risk score as the cutoff. Kaplan Meier survival curve showed that prognosis of patients in low-risk group was significantly better than those in the high-risk group (Figure 5A).

**TABLE 1:** The coef of the IRGPI in the multivariate Cox regression analysis.

id	coef
*FGF18*	1.265
*IDO1*	0.367
*BIRC5*	1.205
*WDR62*	−2.457
*NUMBL*	0.881
*TYRO3*	1.109

Coef, regression coefficient.

Next, we evaluated the predictive ability of the prognostic signature in an independent validation cohort (GSE2748) from the GEO database. GSE2748 contained 2 highly distinct molecular PRCC subclasses. Class 1 was known to have a good prognosis, while the prognosis of Class 2 is poor. The results illustrated in Figure 5B show that Class 2 had a higher risk score than Class 1, indicating that the IRGPI had great value and potentials in accurately predicting patient prognosis (Figure 5B). Finally, we performed qRT-PCR to measure the expression levels of the 6 genes in the IRGPI. Compared to HK-2 cells, *FGF18*, *IDO1*, *BIRC5*, *NUMBL* and *TYRO3* were upregulated in ACHN cells, whereas *WDR62* were repressed in ACHN cells, which was consistent with the risk coefficient of each gene (Figure 5C).

### Molecular characteristics of different immune-related gene prognostic index subgroups

We identified the gene sets enriched between low and high-risk patients, and found that enriched functional pathway showed significant difference between high and low risk score groups (Figure 5D). The gene sets of the IRGPI-high group were enriched in cytokine receptor interaction and ECM receptor interaction, while the gene sets of the IRGPI low group were enriched in energy metabolism.

Next, we analyzed the differences of tumor mutational burden (TMB) in the different IRGPI subgroups (Figure 5E). We found that the most common mutation type was missense mutation, followed by nonsense and frameshift deletions. We then identified the top 20 genes with the highest mutation rates in the IRGPI subgroups and discovered that *TTN* was the most commonly mutated genes, showing mutation rates of over 10% in in both subgroups.

### Immune Characteristics of different immune-related gene prognostic index subgroups

**FIGURE 6:**
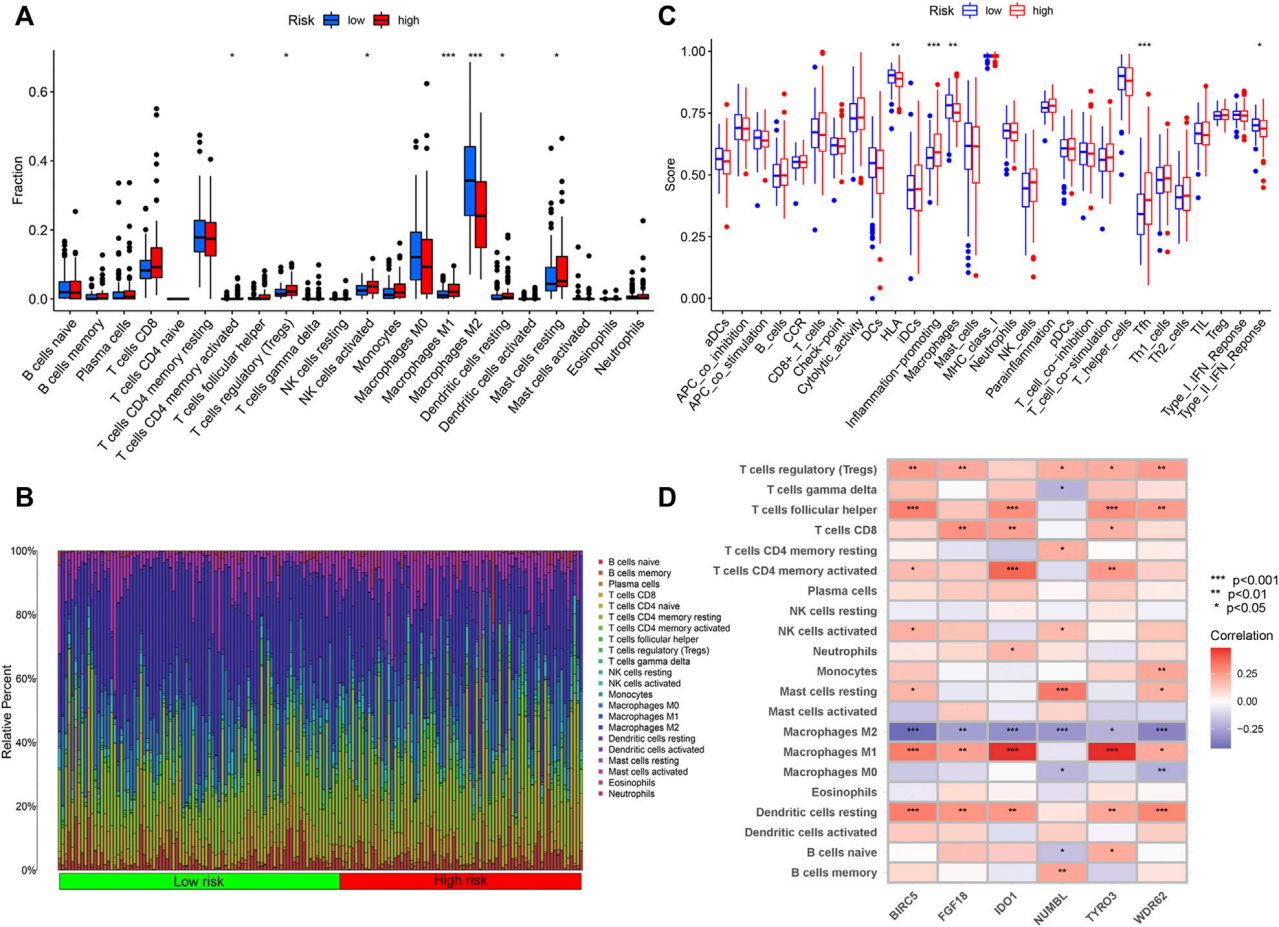
Immune Characteristics of different IRGPI subgroups. **(A)** Correlations of IRGPI with immune cell infiltration. **(B)** Infiltration profiles in the high and low risk groups. **(C)** Correlations of IRGPI with immune function. IRGPI: immune-related gene prognostic index. **(D)** Heatmap of the correlation of the levels of infiltration of the immune cells with 6 risk genes. ***p < 0.001, **p < 0.01, *p < 0.05.

Wilcoxon test was used to compare the distribution of immune cells in different IRGPI subgroups for further exploration of the indicative roles of IPGRI. CIBERSORT was adopted for evaluation of the relative proportion of 22 types of immune cells in all PRCC samples. Activated memory T cells CD4, regulatory T cells, activated NK cells, macrophages M1, resting dendritic cells and resting mast cells infiltration were higher in the high-risk group, while macrophages M2 infiltration were higher in the low-risk group (Figure 6A and Supplementary Figure S1). Immune cell infiltration data of all samples for the high and low risk score groups was showed in Figure 6B. The difference of immune-related function in the tumor immune microenvironment also existed between the high and low risk groups (Figure 6C). We further utilized correlation analysis for 6 risk genes and infiltrating immune cell types to investigate the potential influence of the IRGPI on the immune microenvironment of PRCC (Figure 6D). Additionally, we evaluated the prognostic value of different levels of immune cells and immune function, respectively. The statistically significant variables were showed in Supplementary Figures S2, S3.

### Clinical correlation analysis

**FIGURE 7:**
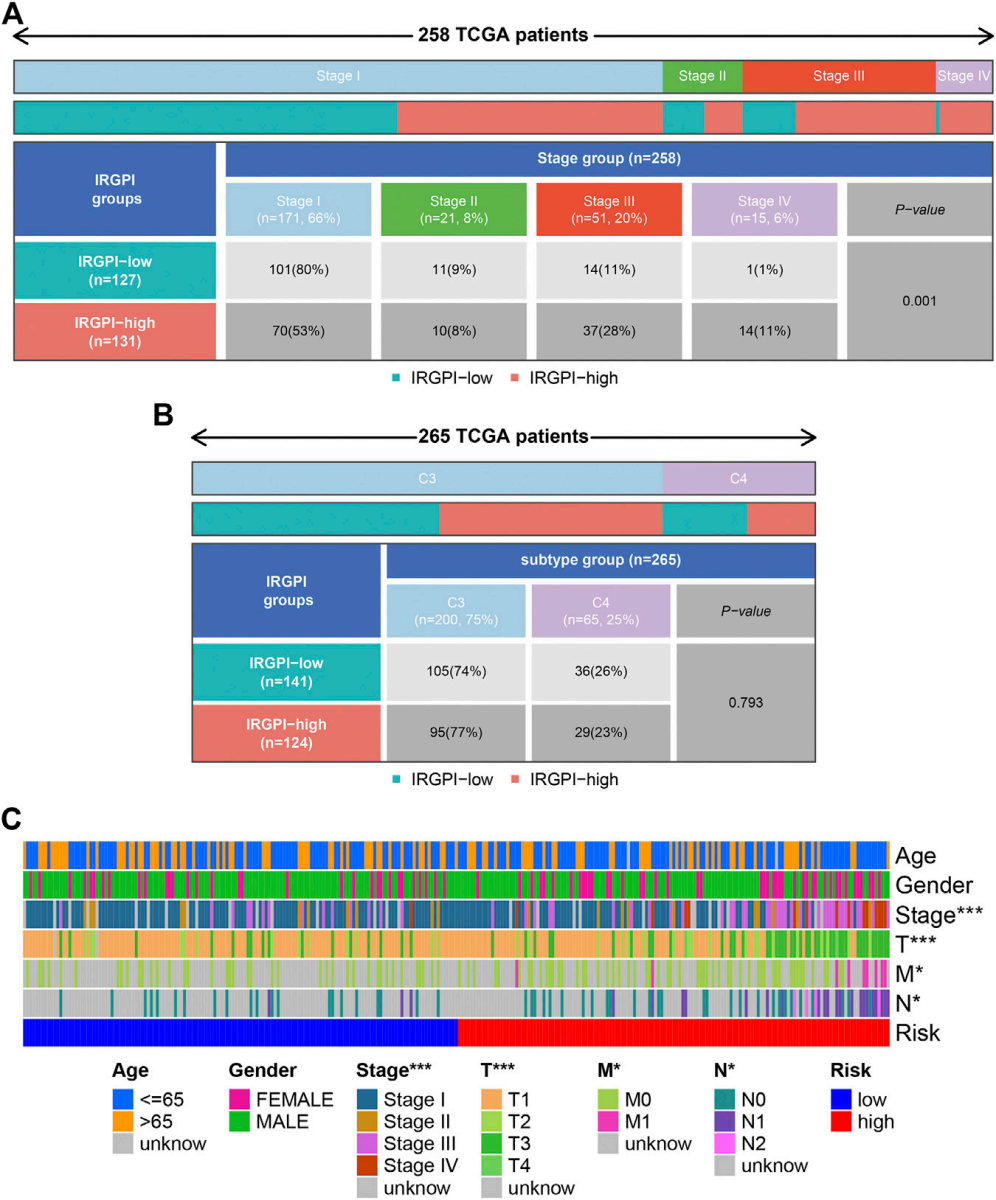
Clinical correlation analysis. Heatmap and table showing the distribution of PRCC pathological stage **(A)** and immune subtypes **(B)** between the IRGPI subgroups. **(C)** Cellular landscape of the relationship between the IRGPI and the different clinical features, including the age, gender, tumor stage, T stage, tumor N stage, and tumor M stage. ***p < 0.001, **p < 0.01, *p < 0.05. IRGPI, immune-related gene prognostic index.

All PRCC samples were further classified according to different pathological stages. We could find from Figure 7A that the number of patients of progressive stage (stage Ⅲ and Ⅳ) in high risk group was significantly higher than those in low risk group; the situation was opposite in the early stage patients (*p* < 0.05). However, the high and low groups were not statistically significant in the distribution of different immunology classification (Figure 7B). Moreover, we used the cellular landscape to assess the clinical value of the model according to the different clinical features of the samples, including the age, gender, tumor stage, T stage, tumor N stage, and tumor M stage (Figure 7C). The results suggested IRGPI showed significant correlation with the tumor’s stage, and could be regarded as a valuable index for predicting the prognosis.

### Evaluation of the immune-related gene prognostic index

**FIGURE 8:**
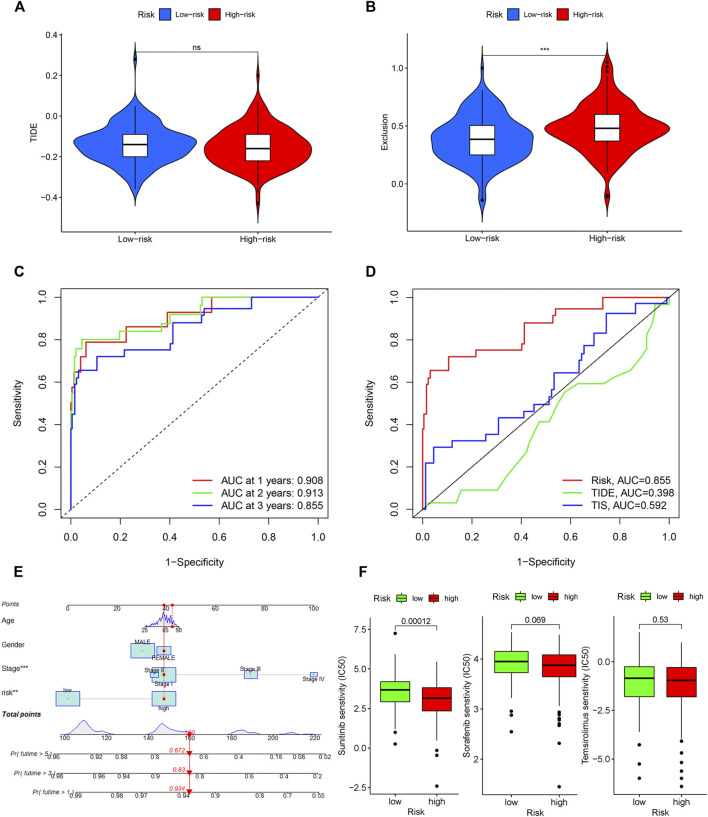
Evaluation of the IRGPI. TIDE **(A)**, and T-cell exclusion **(B)** score in different IRGPI subgroups. ***p < 0.001, **p < 0.01, *p < 0.05. **(C)** Survival-dependent ROC curve analysis of the prognostic value of the IRGPI at 1, 3, and 5 years. **(D)** Survival-dependent ROC curve analysis of the prognostic value of IRGPI, TIS and TIDE at 3 years. TIS, T cell inflamed signature; TIDE, tumor immune dysfunction and exclusion; IRGPI, immune-related gene prognostic index. **(E)** Nomogram for predicting 1, 3 and 5 years overall survival for PRCC patients. **(F)** Box plots of differentially targeted drug sensitivities between two subgroups.

TIDE was used to evaluate the potential clinical efficacy of immunotherapy in different IRGPI subgroups. Higher TIDE prediction score represented a higher potential for immune evasion, indicating that patients were less likely to benefit from ICIs treatment and have a worse outcome (Chen et al., 2021). Our results revealed that the IRGPI increased significantly with low TIDE score, and suggested that the high-risk group patients could benefit relatively less from ICIs therapy and may have a worse outcome (*p* > 0.05, Figure 8A). Besides, the high-risk group patients had a higher T-cell exclusion score (*p* < 0.05, Figure 8B), but there was no significant difference in microsatellite instability (MSI) score and T-cell dysfunction (The results were not shown).

A time-dependent ROC curve was used to assess the prognostic value of IRGPI in the TCGA PRCC cohort. The AUC values of IRGPI at 1, 3, and 5 years were 0.908, 0.913, and 0.855, respectively (Figure 8C). Next, we assessed the predictive value for the prognosis of IRGPI, TIS and TIDE at 3 years. We could find that the AUC for IRGPI were better at 3 years follow-up, suggesting that the predictive value of IRGPI was superior to that of TIS and TIDE (Figure 8D).

### Nomogram and drug sensitivity analysis

To explore IRGPI value for clinical application, we constructed a novel prognostic nomogram to provide a reliable intuitive and quantitative method for predicting the survival of the HCC patients based on the risk scores and clinical features, including age, gender and stage (Figure 8E). The nomogram could effectively predict the predict the survival rates of BLCA patients at 1, 3, and 5-years.

The limited research conducted thus far demonstrates convincingly that sunitinib and sorafenib are the recommended treatment options for those PRCC patient cohort and can prolong the survival time effectively (Tannir et al., 2016; Bergmann et al., 2021). Besides, temsirolimus is also a drug option with high-level evidence (Tsimafeyeu, 2017). Then we studied the role of the IRGPI in predicting the chemosensitivity in PRCC patients. We founded that the high-risk patients were associated with a lower half inhibitory centration (IC50) of chemotherapeutics such as sunitinib (*p* < 0.05), sorafenib (*p* > 0.05), and temsirolimus (*p* > 0.05), indicating that the prognostic index could acted as a potential predictor for chemosensitivity (Figure 8F).

## Discussion

In recent years there has been a remarkable progress in our knowledge regarding the genetic profile of PRCC, which provides the foundation for the development of improved methods for diagnosis, treatment and prevention of this disease. For a long time, using the patient’s own immune system to combat cancer has been considered an attractive therapeutic option, especially in tumors with high immune cell infiltration (Linehan and Ricketts, 2019). Some studies have shown that IL-2 had a profound impact on the development of cancer immunotherapy and had been used in advanced metastatic RCC, resulting in a substantial rate of complete response (Rosenberg, 2014). Besides, PD-1 and its ligand PD-L1 being expressed in a majority of PRCC has resurfaced interest in using immunotherapy as treatment (Chen et al., 2019). The publicly available TCGA data provide potential targeted therapies and invaluable resources for better patient management and treatment of PRCC patients, and could expand our knowledge of etiology mechanism and improve the outcomes of patients with this disease (Liu et al., 2020; Zhou et al., 2020). As multiple oncogenic mutations might co-occur in the same tumor, we needed to identify an IRG signature, which would be promising targets for development of new therapeutic agents.

In this study, we identified differentially expressed immune genes based on the clinical information and transcriptomic data of PRCC from the TCGA cohort. We then subjected those ir-DEGs to GO and KEGG analyses, which revealed that they were mainly enriched for neutrophil and leukocyte-related biological behavior and the regulation of immune effector process. The gene modules closely related to the tumorigenesis of PRCC were screened by WGCNA, and genes associated with prognosis of patients with PRCC were further screened with univariate Cox regression. Subsequently, 6 of 29 screened genes, including *FGF18*, *IDO1*, *BIRC5*, *WDR62*, *NUMBL* and *TYRO3*, were used to construct the prognostic index of immune-related genes that was used to calculate the risk scores of PRCC patients. Overexpression of FGF18, relating to the embryonic and postnatal development of cartilage, hair, and vasculature, plays an important role in the process of invasion and metastasis of several neoplasms, including hepatocellular carcinoma (Guo et al., 2018), gastric cancer (Zhang et al., 2019a), and colon cancer (Shimokawa et al., 2003), while high expression of FGF18 correlates with a good prognosis in ccRCC patients (Yang et al., 2020). Some studies have shown that *IDO1* was associated with a poor prognosis of PRCC, and *IDO1* inhibitor may be effective for treating sarcomatoid/rhabdoid RCC (Zhang et al., 2019b; Kiyozawa et al., 2020). Accumulating evidence shows that *BIRC5* (also known as survivin) is closely related to tumor progression, tumor progression, tumor recurrence, chemotherapy resistance and poor prognosis (Liu et al., 2015; Zhang et al., 2019c), and high expression levels of *BIRC5* predicted a poor outcome for ccRCC patients (Parker et al., 2006). *TYRO3* is a key part of the tumor-associated macrophage (TAM) receptor-ligand complex, which are implicated in several hallmarks of cancer progression and involves in the acquisition of the resistance to sunitinib in renal cell carcinoma (Pinato et al., 2016). The research about the functional roles of *NUMBL* and *WDR62* in renal cell carcinoma is seldom. *Numbl*, a developmentally-regulated polarity protein, becomes subcellularly deregulated and over-expressed in various human cancers (Vaira et al., 2013). Sugita et al. (2019) demonstrated that the downregulation of *WDR62* induced the apoptosis and inhibited the viability of bladder cancer cells.

Based on these scores, all patients in the TCGA cohort were divided into high- and low-risk groups. Survival analysis indicated that high risk patients had an aggressive clinical course with poor prognosis, which was consistent with result of validation set. Moreover, the tendency of component gene expression was the same as risk coefficient (positive or negative) in the IRGPI, confirming the role of these genes in PRCC. GSEA revealed that the two subgroups differed markedly in respect of the enriched gene sets. AS one of the most enriched gene set in high-risk group, assembly of the cytokine–receptor complex activates intracellularly associated JAK/STAT signaling, the Akt and Erk pathways as well as other signaling networks in the typical cytokine signaling pathway (Ihle et al., 1995; Platanias, 2005; Spangler et al., 2015). Cytokine-receptor interaction may be critical in determining the effects of inflammation in the development of the disease (Qian et al., 2019). The low-risk group enriched gene set we were most interested in is butanoate metabolism. Butyrate is one of the three most common short-chain fatty acids, which exerts many renoprotective properties, such as anti-inflammation, anti-atherosclerosis, anti-oxidative functions (Sun et al., 2018; Wu et al., 2020a). Besides, Chang et al. (2014) found that Butyrate is involved in the maintenance of intestinal epithelial cells and plays a significant role in regulating intestinal immune tolerance to antigens (Wang et al., 2020). Mutation diversity analysis demonstrated that the most common mutation type in the two subgroups was missense mutation, and the most common mutant gene in the two subgroups was *TTN*. The distribution order of *TTN* coding sequence variants associated with human conditions were as follows: nonsense mutations, frameshifts, missense variants and splice-site variants (Chauveau et al., 2014). Previous studies reported that *TTN* mutation was associated with better response to immune checkpoint blockage in solid tumors, but the potential mechanisms were still unclear. It was reported that if TMB is larger, the cancer cell is more mutated, and it is easier for immune cells to recognize and kill it (Miao et al., 2018), which was in conformance with this result that TMB was higher in PRCC patients with low-IRGPI scores.

The correlation between IRGPI subgroups and tumor-infiltrating immune cells was analyzed to reflect on the status of the immune microenvironment. Treg cells abundantly infiltrate into tumor tissues and are often associated with poor prognosis in cancer patients (Tanaka and Sakaguchi, 2019), which supports our findings. Surprisingly in this study, macrophages M1 infiltration was significantly increased in the high-risk group, while macrophages M2 infiltration was higher in the low-risk group. However, the trend of immune cell infiltration of our study was consistent with in a previous study (Zhou et al., 2020), and could be explained by several research findings to some degrees. Recent studies have found significant differences among monocytes or macrophages from distinct tumors, and other investigations have explored evidence that specific localization of TAMs and differences in the tumor microenvironment may also impact their inter- and intra-tumoral heterogeneity and how they affect tumor growth (Park et al., 2016; Wu et al., 2020b). Previous research in ccRCC showed that the abundance of CD8^+^ T cells was positively correlated with the abundance of Tregs and T cells follicular helper, and negatively correlated with the abundance of M2 macrophages, which provides a theoretical support for our research results (Pan et al., 2020). Further studies are required to understand how the macrophage phenotype changes in different tumor microenvironments and how this affects tumor growth and spread.

By Integrating with tumor stage, we found that advanced stage was associated with the IRGPI-high subgroup, while early stage with the IRGPI-low subgroup. Moreover, cellular landscape showed that IRGPI was a potential factor which forecasting the clinical outcome, including tumor stage, T stage, tumor N stage, and tumor M stage. To gain further biological insight into the potential clinical efficacy of immunotherapy in different IRGPI subgroups, the relationship between IRGPI and the mechanisms of immune escape was explored in PRCC patients. It has been reported that the TIDE prediction score was correlated with T cell dysfunction in CTL-high tumors and T cell exclusion in CTL-low tumors and thus represents two different mechanisms of immune escape (Chen et al., 2021). In our study, IRGPI-high patients had lower TIDE score (*p* > 0.05) and higher T cell exclusion score (*p* > 0.05) than IRGPI-low patients. Thus, we can speculate that the lower ICI response of IRGPI-high patients may be principally due to immune evasion via T cell exclusion. Finally, time-dependent ROC curve was used to evaluate the prognostic value of IRGPI, TIS and TIDE at 3 years. It has been confirmed that the TIDE score predicts the outcome of melanoma patients treated with first-line anti-PD1 or anti-CTLA4 antibodies more accurately than other biomarkers, such as PD-L1 level and mutation load (Jiang et al., 2018). TIS, developed by NanoString Technologies, was a clinical-grade assay, which can provides both quantitative and qualitative information about the tumor microenvironment (TME) and predicting response to anti-PD-1/PD-L1 agents (Seiwert et al., 2015). This study demonstrated that the IRGPI score had a better prognostic value and might serve as a better predictor of OS compared with TIDE and TIS. Base on the IRGPI and other clinical parameters that were generally believed to have a certain impact on the prognosis of PRCC, we constructed a nomogram to predict the PRCC patients’ overall survival. Further, our research revealed that the IRGPI could act as a potential predictor for sensitivity to chemotherapeutical agents, including sunitinib, sorafenib, and temsirolimus. However, the lack of experimental verification in the pathological specimens was a significant deficiency of our study.

In conclusion, we identify an IRGPI which is closely related to the immune microenvironment and provides a comprehensive and systematic analysis of distinct phenotypic and molecular portraits in the recognition, surveillance, and prognosis of PRCC. Finally, IRGPI could be used as a potential biomarker to evaluate the response and efficacy of immunotherapy in PRCC.

## Data Availability

The original contributions presented in the study are included in the article/Supplementary Material, further inquiries can be directed to the corresponding authors.

## Supplementary material

SUPPLEMENTARY FIGURE S1 The correlation of the infiltrating immune cells with the IRGPI.

SUPPLEMENTARY FIGURE S2 KM survival analysis of PRCC patients in different subgroups with different levels of immune cell infiltration.

SUPPLEMENTARY FIGURE S3 KM survival analysis of PRCC patients in different subgroups with different levels of immune function.
