# Corrigendum: Identification of an immune-related gene prognostic index for predicting survival and immunotherapy efficacy in papillary renal cell carcinoma

**DOI:** 10.3389/fgene.2024.1440802

**Published:** 2024-06-26

**Authors:** Dongshan Chen, Chen Zhang, Yuanwei Zang, Wei Wang, Jiandong Zhang

**Affiliations:** ^1^ Department of Urology, Beijing Chaoyang Hospital Affiliated Capital Medical University, Beijing, China; ^2^ School of Life Science and Engineering, Handan University, Handan, China; ^3^ Department of Urology, Qilu Hospital of Shandong University, Jinan, China; ^4^ Department of Urology, Shanxi Bethune Hospital, Shanxi Academy of Medical Sciences, Taiyuan, China; ^5^ Department of Urology, Tongji Hospital, Tongji Medical College, Wuhan, China

**Keywords:** immune cell infiltration, immune-related genes, papillary renal cell carcinoma, prognostic index, immunotherapy

In the published article, there was an error in the **Affiliations**. The corrected affiliations and corresponding author attributions appear below.

“Dongshan Chen^1,‡^, Chen Zhang^2,‡^, Yuanwei Zang^3,‡^, Wei Wang^1,^*^,†^ and Jiandong Zhang^1,4,5,^*^,†,‡^”.

“^1^Department of Urology, Beijing Chaoyang Hospital Affiliated Capital Medical University, Beijing, China

^2^School of Life Science and Engineering, Handan University, Handan, China

^3^Department of Urology, Qilu Hospital of Shandong University, Jinan, China

^4^Department of Urology, Shanxi Bethune Hospital, Shanxi Academy of Medical Sciences, Taiyuan, China

^5^Department of Urology, Tongji Hospital, Tongji Medical College, Wuhan, China”.

The authors apologize for these errors and state that this does not change the scientific conclusions of the article in any way. The original article has been updated.

