# Supplementary figures and images for: Identification of an immune-related gene prognostic index for predicting survival and immunotherapy efficacy in papillary renal cell carcinoma
Source: Front Genet. 2022 Aug 29;13:970900. doi: 10.3389/fgene.2022.970900 (PMC9499392; doi:10.3389/fgene.2022.970900)

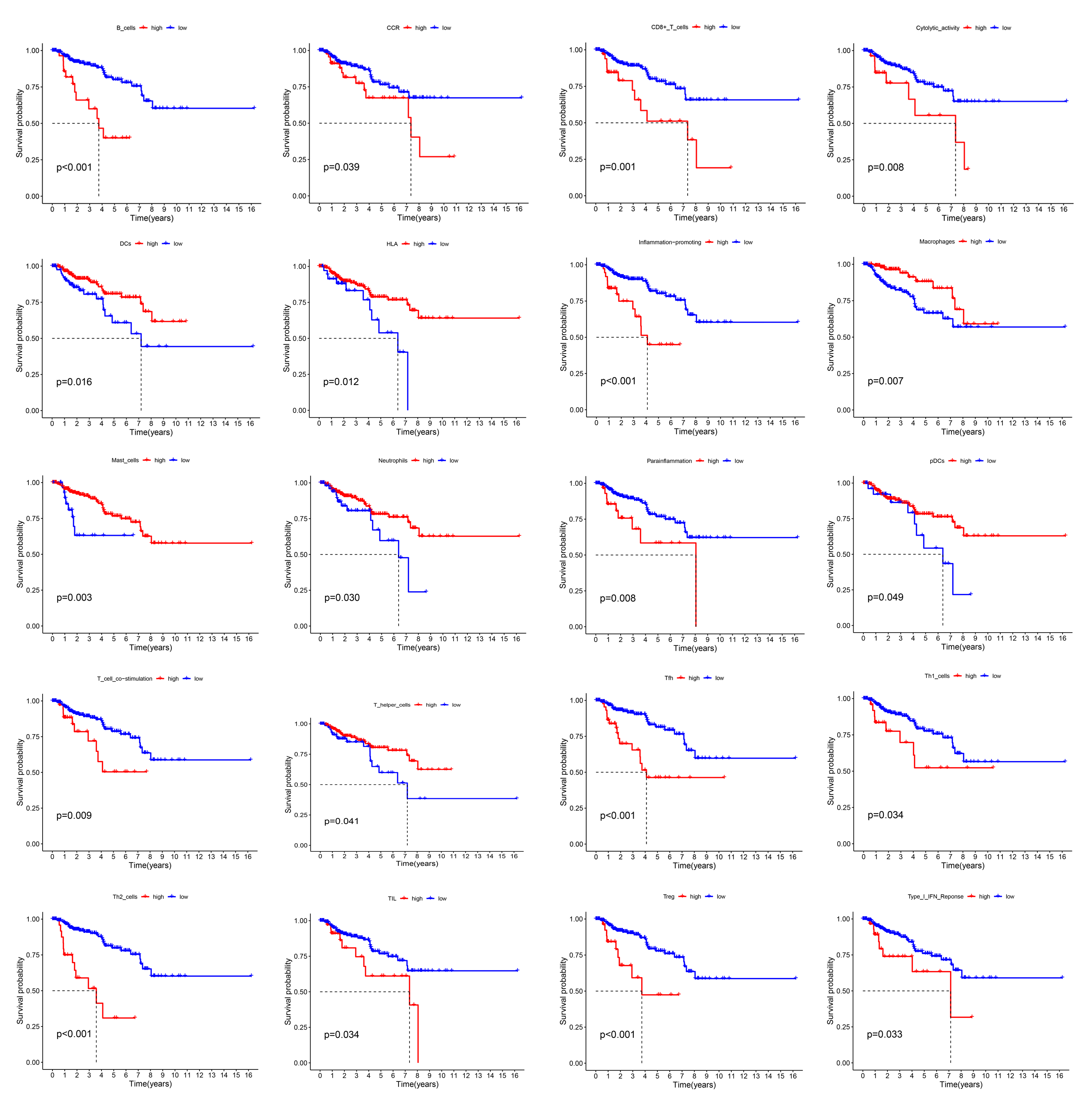

Supplement: Supplementary file 1 [file Image3.TIF]

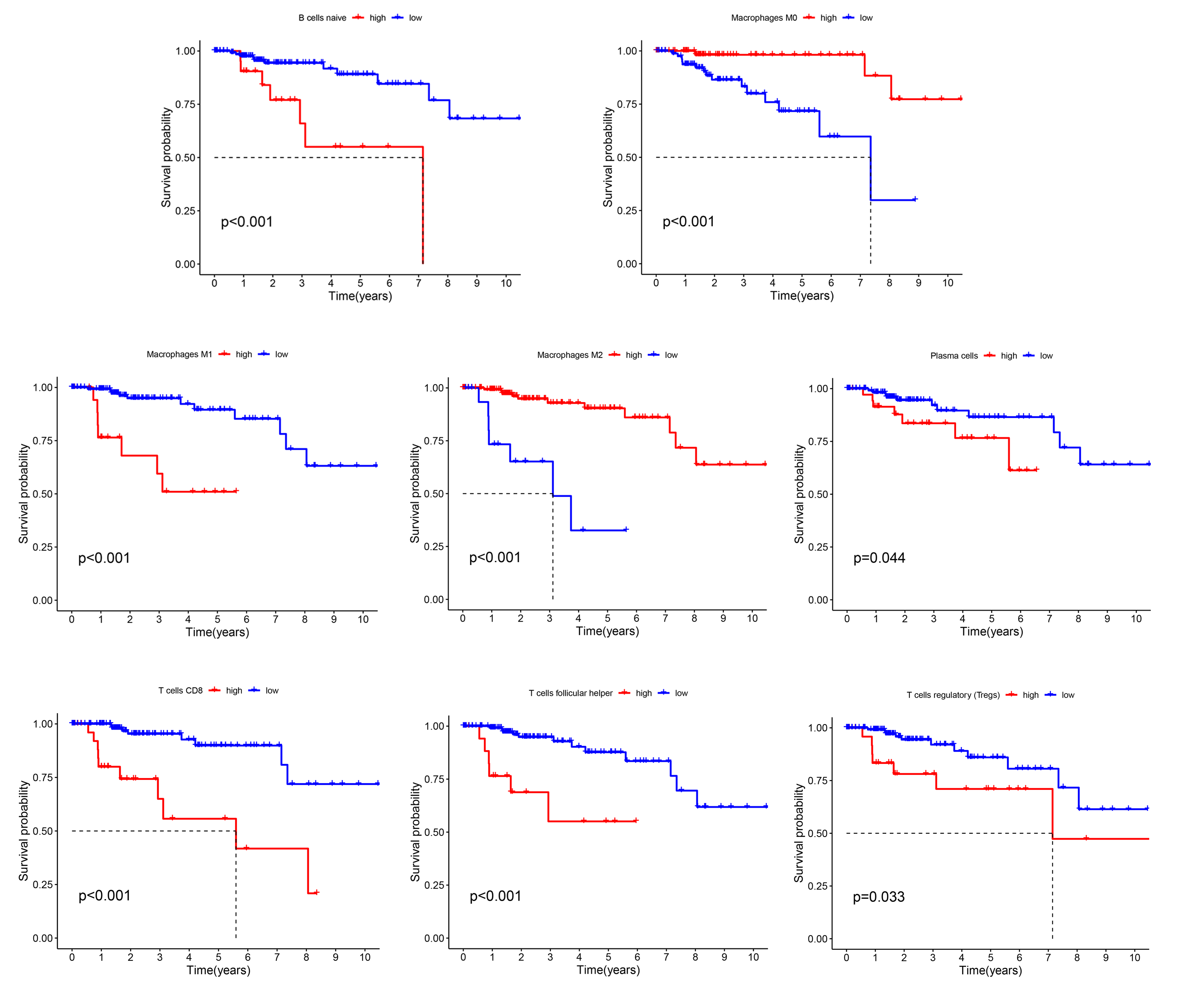

Supplement: Supplementary file 2 [file Image2.TIF]

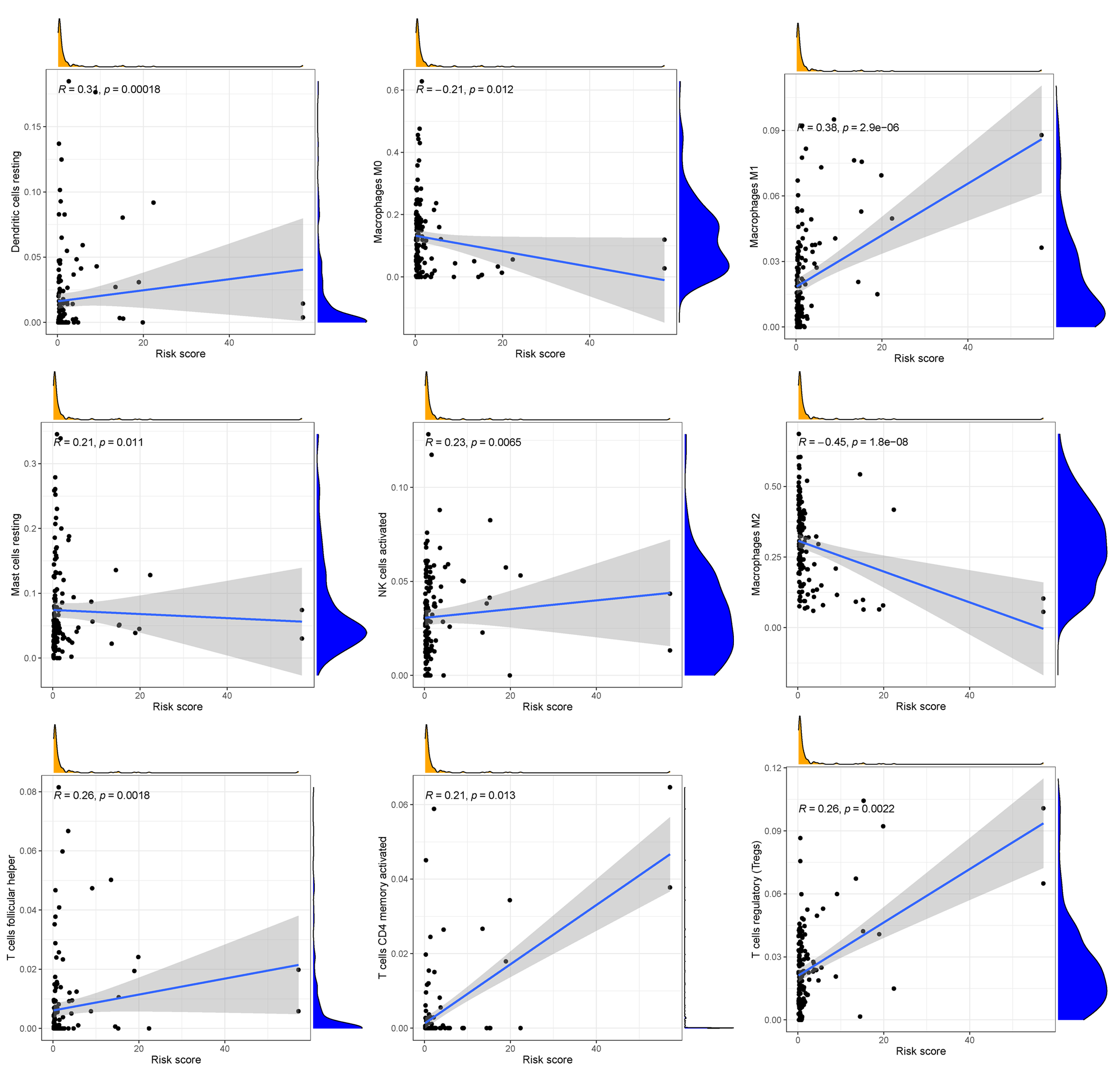

Supplement: Supplementary file 3 [file Image1.TIF]
