# Supplementary material for: Identification of an immune-related gene prognostic index for predicting survival and immunotherapy efficacy in papillary renal cell carcinoma
Source: Front Genet. 2022 Aug 29;13:970900. doi: 10.3389/fgene.2022.970900 (PMC9499392; doi:10.3389/fgene.2022.970900)
Supplement: Supplementary file 4 [file Table2.DOCX]

**Supplementary Table S1** Primer sequences used in this study

| Gene | Forward | Reverse |
| --- | --- | --- |
| *FGF18* | TGCTTCCAGGTACAGGTGCT | GCTGCTTACGGCTCACATCG |
| *IDO1* | GATCATCTCACAGACCACAAGTCACAG | CTTGGAGAGTTGGCAGTAAGGAACAG |
| *BIRC5* | CGCATCTCTACATTCAAG | ATGT TCCTCTCTCGTGAT |
| *WDR62* | AGT AAC CCC CAG CTT CCA GAG | CTG CCC TGT AAC AGA CCA AGT G |
| *NUMBL* | ATGAACAAGTTACGGCAGAGC | CTACAGTTCAATCTCGAATGT |
| *TYRO3* | GAGGATGGGGGTGAAACC | ACTGTGAAAAATGGCACACCT |
| *GAPDH* | GATGGAGGAGGCTCAGCA | CTCAGCCAATGGGACCTG |
